# Myxoid Liposarcoma: Prognostic Factors and Metastatic Pattern in a Series of 148 Patients Treated at a Single Institution

**DOI:** 10.1155/2018/8928706

**Published:** 2018-05-16

**Authors:** Francesco Muratori, Leonardo Bettini, Filippo Frenos, Nicola Mondanelli, Daniela Greto, Lorenzo Livi, Alessandro Franchi, Giuliana Roselli, Maurizio Scorianz, Rodolfo Capanna, Domenico Campanacci

**Affiliations:** ^1^Divisione di Ortopedia Oncologica e Ricostruttiva Ospedale, Azienda Universitaria Ospedaliera Careggi Firenze, Firenze, Italy; ^2^Dipartimento di Radioterapia Azienda Ospedaliera Universitaria Careggi, Firenze, Italy; ^3^Dipartimento di Ricerca Traslazionale e delle Nuove Tecnologie in Medicina e Chirurgia, Università di Pisa, Pisa, Italy; ^4^Divisione di Radiologia Ospedale, Azienda Universitaria Ospedaliera Careggi Firenze, Firenze, Italy

## Abstract

**Objectives:**

The authors reported a retrospective study on myxoid liposarcomas (MLs), evaluating factors that may influence overall survival (OS), local recurrence-free survival (LRFS), metastasis-free survival (MFS), and analyzing the metastatic pattern.

**Methods:**

148 MLs were analyzed. The sites of metastases were investigated.

**Results:**

Margins (*p* = 0.002), grading (*p* = 0,0479), and metastasis (*p* < 0,0001) were significant risk factors affecting overall survival (OS). Type of presentation (*p* = 0.0243), grading (*p* = 0,0055), margin (*p* = 0.0001), and local recurrence (0.0437) were risk factors on metastasis-free survival (MFS). Authors did not observe statistically significant risk factors for local recurrence-free survival (LRFS) and reported 55% extrapulmonary metastases and 45% pulmonary metastases.

**Conclusion:**

Margins, grading, presentation, local recurrence, and metastasis were prognostic factors. Extrapulmonary metastases were more frequent in myxoid liposarcoma.

## 1. Introduction

Liposarcoma is one of the most common sarcomas found in adults [1, 2] and it can be defined as a mesenchymal malignancy characterized by adipocyte differentiation. Different forms of liposarcoma are described: atypical lipomatous tumor/well differentiated (ALT/WD), dedifferentiated liposarcoma (DDLs), myxoid liposarcoma (MLs), and pleomorphic liposarcoma (PLs) [2–5].

Myxoid liposarcoma is the second most common subtype (MLs). It accounts for 15–20% of liposarcomas and represents about 5% of all soft tissue sarcomas in adults. Histologically MLs show a continuous spectrum of lesions with low grade forms and others poorly differentiated round cells forms [2].

MLs presents the recurrent translocation *t*(12;16)(q13;p11) that results in* FUS-DDIT3 *gene fusion, present in >95% of cases. In the remaining cases, a variant *t*(12;22)(q13;q12) is present in which* DDIT3 *(also known as* CHOP*) fuses instead with* EWSR1*, a gene that is highly related to* FUS.* They have a peak incidence in the fourth and fifth decade of life, in particular on the lower extremities and buttock [2, 6, 7].

Another feature that distinguishes the MLs than other liposarcomas is the tendency to metastasize in unusual regions correlated to worst prognosis and more precisely where fat tissue is present as the trunk, extremities, bone, retroperitoneal site, the chest wall, the pleura, and pericardium [8–11].

Factors affecting the prognosis in MLs include age at diagnosis, tumor size, tumor grade, depth of tumor, and surgical margins [12–16]. Differentiation, necrosis, mitotic rate, proliferation index (MIB-1, Ki-67 immunostain), and overexpression of P53 represent morphological prognostic factors in MLs [12, 13, 16]. Surgical excision with or without radiation therapy is the treatment of choice in the localized MLs. Chemotherapy is generally reserved for patients with high risk disease such as high grade, deep sited tumor, tumor size > 5 cm, and positive surgical margins.

The aim of our retrospective study was to evaluate factors that may influence overall survival (OS), local recurrence-free survival (LRFS), and metastasis-free survival (MFS) in a series of 148 patients with MLs treated in a single center. We analyzed the metastatic pattern of MLs and the propensity to give extrapulmonary metastases to define a proper clinic and imaging pathway.

## 2. Materials and Methods

We retrospectively reviewed histological and clinical records of 148 patients treated between 1994 and 2015. The mean age was 49 years (16–82), 142 (96%) liposarcomas localized in the limbs and 6 (4%) in the trunk.

All data collected included patient characteristics (age, gender), tumor characteristics (site, size, clinical symptoms, stage, and histology), the diagnostic and therapeutic procedures (type of biopsy, type of surgery, margins, neoadjuvant, and adjuvant therapy), and clinical outcome.

The data were obtained from the patient's medical records. Local recurrence and distant metastasis after treatment were recorded. Each patient underwent anamnestic collection of his medical history, physical examination, and routine blood tests; electrocardiogram and chest X-ray were obtained. Considering that X-ray or CT were not useful to identify the features and the edges of the primary tumor, MRI was performed in most patients. MRI was particularly useful in defining certain characteristics such as homogeneity, necrosis, hemorrhagic areas, the local spread of the disease (size), and tumor stages. Chest CT scan, bone scan, or PET (from 2009) was performed preoperatively.

At diagnosis, all patients had a localized soft tissue sarcoma in absence of metastases.

Histological diagnosis was confirmed by open incisional biopsy, ultrasound needle biopsy, or previous inadvertent excision performed at other centers. All available histologic slides were reviewed and tumors were graded according to WHO 2013 classification of soft tissue sarcomas [2]. High grade (“round cell”) areas were characterized by solid sheets of back-to-back primitive round cells with a high nuclear to cytoplasmic ratio, with no intervening myxoid stroma [2]. If these areas represented more than 5% of the tumor, this was considered as high grade. FISH for* DDIT3 *was performed in dubious forms of high grade MLs for the differential diagnosis with other soft tissue sarcomas.

Following the initial work-up the surgical approach was the main treatment that attempts to get wide margins. When the tumor was adjacent to critical structures such as nerves, blood vessels, or bones, a planned marginal surgery has been accepted.

Radiotherapy (RT) in preoperative or postoperative setting was performed in patients with high grade disease or tumor size > 5 cm and deep sited tumors or in case of close/positive margins.

External beam radiotherapy was delivered with 6–10 MeV photons; Gtv (Gross Tumor Volume) was obtained contouring the surgical bed or the gross tumor in case of preoperative RT on T1 weighted MRI images, CTV (clinical target Volume) derived from an expansion of 1.5 cm radially, and 4 cm longitudinally from the GTV, and finally 0.5 cm were added to the CTV to obtain the PTV (planning target volume). There was a total dose of 50 Gy and 60 Gy in preoperative and postoperative setting, respectively.

A standard fraction schedule was used: 2 Gy per fraction, 5 days a week.

Chemotherapy was performed in patients with more than two of these unfavourable prognostic factors: high grade disease, tumor size > 5 cm, deep sited tumors, and positive surgical margins. Chemotherapy consisted of three or five cycles of epirubicin (60 mg/m2, Days 1-2) and ifosfamide (3 g/m2, Days 1–3) administered every 21 days.

The patients were followed every 3 months for the first 2 years, every 4 months during 3rd year, every 6 months for 4th-5th years, and annually from 6th to 10th year.

The statistical analysis was performed with MedCalc software version 16.8.4. Values of *p*≤ 0,05 were considered statistically significant. All variables were analyzed for their impact on overall survival, local recurrence-free survival, and metastasis-free survival with a follow-up of 5 and 10 years. In univariate analysis of the overall survival estimates, local recurrence-free survival and metastasis-free survival were calculated according to the method of Kaplan-Meier.

The comparison of survival curves calculated was performed by the log-rank test media. The hazard ratios and confidence intervals (95%) were calculated using the Cox hazard test.

## 3. Results

Our data included 103 (70%) primitive liposarcomas, 26 (17%) local recurrences of primitive liposarcoma, and 19 (13%) radicalizations of liposarcoma treated elsewhere. The locations were the lower extremities in 129 (87%) cases, the upper limb in 13 (9%) cases, and trunk in 6 (4%) cases. Specifically 5 (3%) liposarcomas were localized at the muscles of the shoulder, 3 at the arm, 5 at the elbow and distal to the elbow, 10 in pelvic muscles, 76 in the thigh, and 43 in the knee and distal to the knee. Six liposarcomas were localized in the muscle of the trunk. The preoperative MRI showed size > to 10 cm in 47 (32%) patients, between 5 and 10 cm in 67 (45%) patients, and <5 cm in 34 (23%) patients (Table 1).

100 (68%) tumors were classified low grade (<5% round cells) and 48 (32%) high grade (>5% round cells).

At the final histology 105 (71%) MLs were treated with radical or wide surgery, 41 (28%) with marginal surgery, and 2 (1%) with intralesional excision. The preoperative radiotherapy was performed in 41 MLs (14 cases with size > 10 cm, 18 cases between 5 and 10 cm, and 9 cases with dimensions < 5); the postoperative radiotherapy was performed in 63 patients (14 < 5 cm, 32 between 5 and 10 cm, and 17 > 10 cm) of which 17 patients had marginal or compromised margins at histological examination and in 30 patients with high grade MLs (Table 2).

Chemotherapy was administered in 45 MLs patients with aggressive histological type, 25 neoadjuvant chemotherapy, and 29 postoperative chemotherapy (Table 2).

The average follow-up was 73 months (range 6–257); 76 patients had a greater than 5-year follow-up.

## 4. Local Recurrence

We observed 15 (10%) local recurrences with mean free interval of 29 months (range 1–81 months).

Eight MLs treated with radical or wide excision developed local recurrence, 3 with size > 10 cm, 3 with size > 5 cm, and only 2 with sizes < 5 cm. A patient with local recurrence underwent amputation for involvement of neurovascular bundle, six patients were treated with excision, and one patient was lost.

Seven MLs treated with marginal excision developed local recurrence, 4 with size > 10 cm, 2 with size > 5 cm, and 1 with size < 5 cm. Five local recurrences were treated with excision and 2 with amputation for involvement of neurovascular bundle.

No patients treated with intralesional surgery developed local recurrence.

We did not observe statistically significant risk factors for the local recurrence-free survival (LRFS) (Table 4).

LRFS was 89% at 5 years and 86% at 10 years.

## 5. Metastasis

Twenty MLs (14%), 7 MLs treated with wide resection and 13 with marginal surgery, developed metastases. The sites of metastases were 9 lung, 2 liver, 5 spine, 1 chest wall, 1 peritoneum, 1 kidney, and 1 dorsal soft tissue

One patient treated with intralesional excision died after 3 months, while one patient with MLs (size > 5 cm) treated with intralesional excision and postoperative radiation therapy has not developed local recurrence and metastases after 142 months of follow-up.

Margins (*p* = 0.0001), grading (*p* = 0,0055) (Figure 4), type of presentation (*p* = 0,0243) (Figure 6), and local recurrence (*p* = 0,0437) (Figure 5) are risk factors on metastasis-free survival (MFS) (Table 5).

Five MLs with local recurrences developed distant metastases.

MFS was 85% at 5 years and 82% 10 years.

## 6. Overall Survival

**Figure 1:**
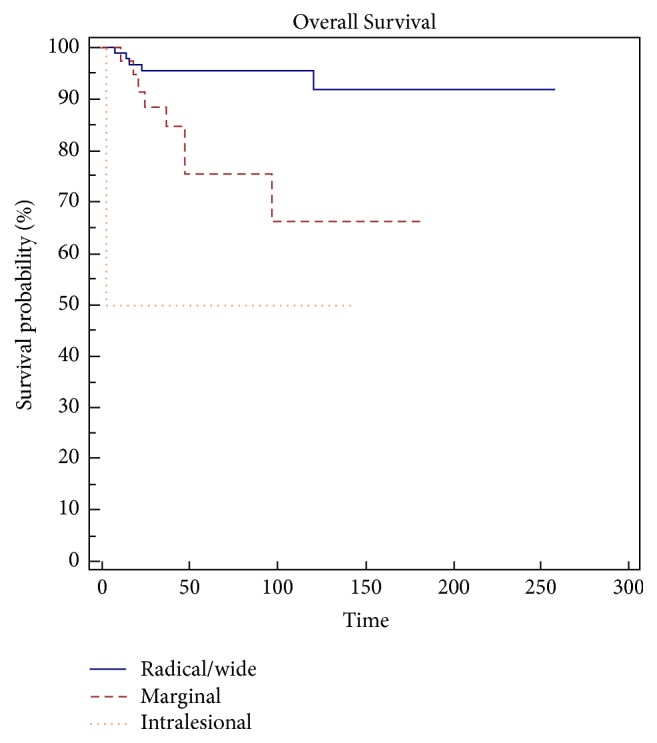
Margins represent a significant risk factor (*p* = 0,002) in overall survival (OS).

**Figure 2:**
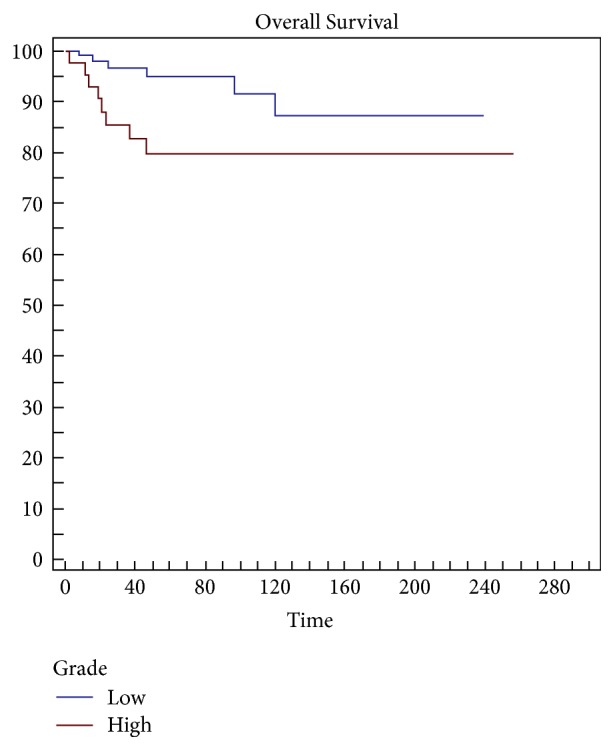
Grading is a significant risk factor (*p* = 0,0479) in overall survival (OS).

**Figure 3:**
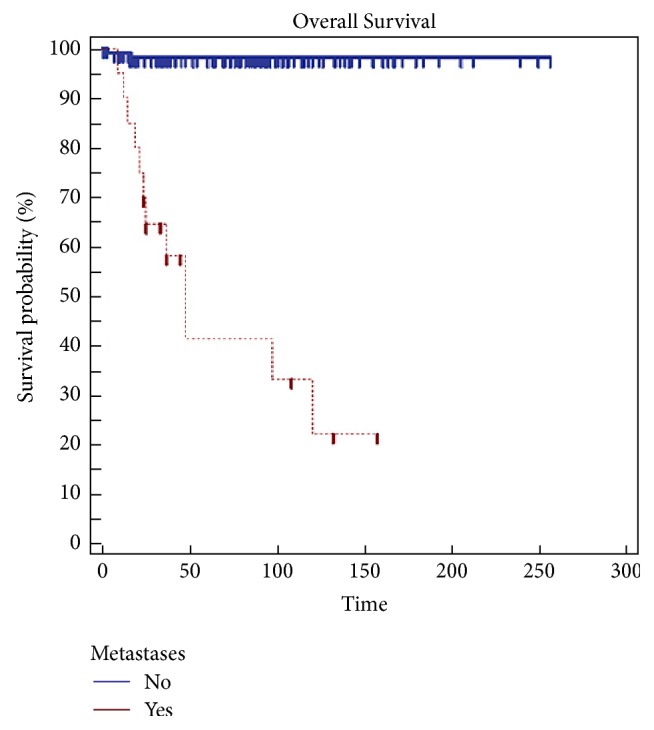
Metastasis is a high significant risk factor (*p* < 0,0001) in overall survival (OS).

**Figure 4:**
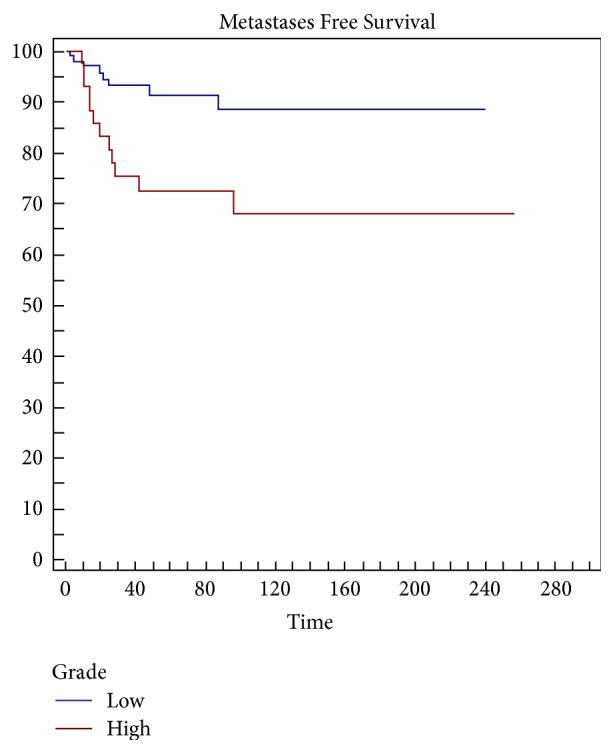
Grading is a significant risk factor (*p* = 0,0055) in metastasis-free survival (MFS).

**Figure 5:**
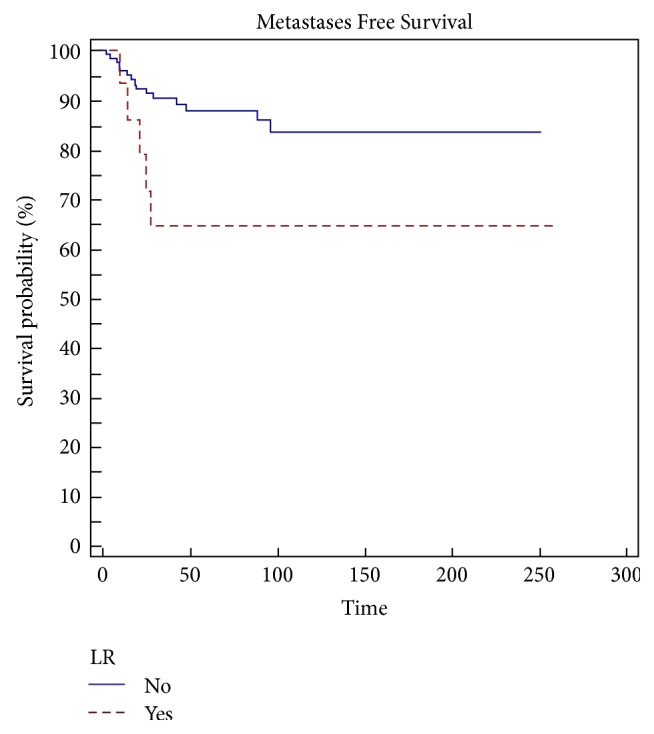
Local recurrence is a significant risk factor (*p* = 0,0437) in metastasis-free survival (MFS).

**Figure 6:**
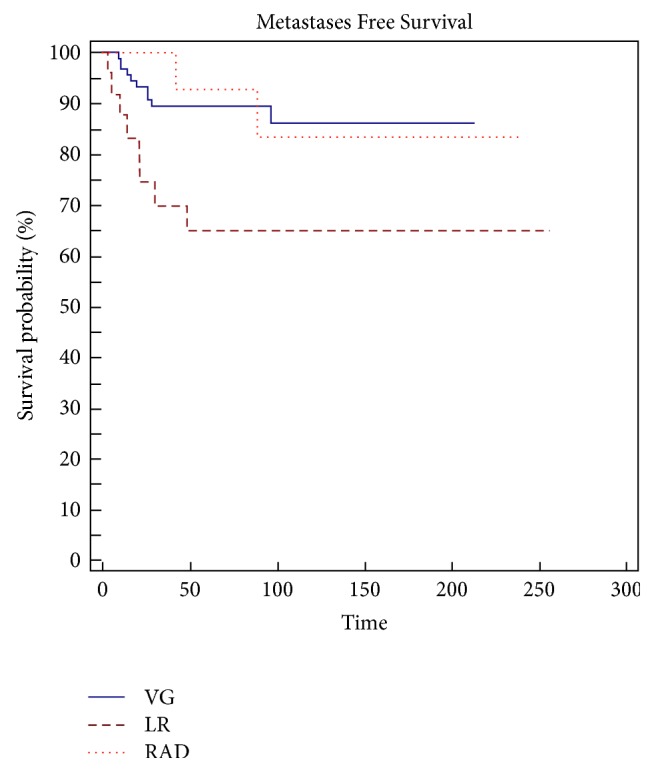
Presentation is a significant risk factor (*p* = 0,0243) in metastasis-free survival (MFS).

Statistical analysis indicates margins (*p* = 0.002) (Figure 1) and grading (*p* = 0.0479) (Figure 2) are a risk factor on overall survival (OS) and the appearance of metastases is a highly significant Factor (*p* < 0.0001) (Figure 3) (Table 3).

OS was 90% at 5 years and 85%, respectively, at 10 years.

## 7. Multivariate Analysis

In multivariate analysis for MFS only the margins (*p* = 0.0004) was statistically significant, unlike the type of presentation (*p* = 0.0906) and the event local recurrence (*p* = 0.0821). In the multivariate analysis for OS only metastasis was statistically significant (*p* < 0,0001), unlike margins (*p* = 0,1039).

## 8. Discussion

The study reports the outcome in terms of recurrence-free survival, metastasis-free survival, and overall survival, in a series of 148 patients with MLs diagnosed and treated in a single center over the last 21 years.

Limb salvage with wide margin is the main treatment in soft tissue sarcomas surgery. Amputation is reserved only when neurovascular bundle is involved, in cases of severe tissue impairment caused by radiotherapy and finally in unsolvable postsurgical infectious complications. Our results showed that surgical margins had an impact on metastasis-free survival (MFS) and overall survival (OS) while local recurrence-free survival (LRFS) was not correlated with margins. Inadequate surgical margins increased the risk to develop metastasis (*p* = 0,0001) affecting negatively OS (*p* = 0.002), according to other reported series [12, 13, 17, 18]. Surgical excision should be carefully planned by experienced surgeons considering the areas in proximity of vascular structures, nerves, and bone [19, 20]. The treatment of MLs in facilities not specialized in cancer care is an important risk factor for local recurrence. Lemeur reported 23% of local recurrence in a series with six patients treated initially in nonspecialized centers, including 4 managed with intralesional excision; only one had a preoperative MRI and no patient underwent preoperative biopsy [14], stressing the importance of surgical planning in agreement with other authors [12, 13, 17, 18]. Engström et al. reported a 47% recurrence for tumors operated in nonspecialized setting [20]. Chandrasekhar et al. reported 59% of local recurrences on 363 cases treated inadequately [21]. This finding is also confirmed by our data: local recurrence of tumors treated in nonspecialized center in cancer care had a higher risk to develop distant metastases (*p* = 0.0243) (Table 5). In our series we observed 15 recurrences (10.1%) in 8 MLs treated with wide and in 7 with marginal surgery. Local recurrence rate was lower compared to 14% observed by Mayo Clinic group [22] and 21.7% at 5 years observed by Fiore et al. [13]. The low rate of local recurrence in our series can be explained by the fact that 70.2% of patients received prior postoperative radiotherapy. Accordingly Guadagnolo et al. observed 3% of local recurrences in 127 MLs treated with preoperative or postoperative radiation therapy [23]. It was postulated that the effectiveness of radiation therapy in myxoid liposarcomas is related to radiosensitivity of the delicate blood supply, characteristic of this tumor [24]. Hannibal et al. observed a very low rate of local recurrence (4%) in patients with purely myxoid liposarcoma (low grade) treated with wide margins. For these patients, the role of radiation therapy appears more questionable [17].

In several series, the proportion of round cell and the histologic grade represent a prognostic factor influencing the overall survival. This was confirmed by our data: overall survival was 95% at 5 years and 87% at 10 years for MLs with round cells < 5% and 80% at 5 years and 80% at 10 years for MLs with round cells > 5%. Fiore et al. reported 93% overall survival for patients with MLs including round cell forms [13]. Haniball et al. reported a dramatically worse 5-year survival of 58% [17] highlighting that round cell > 5% increases the risk of local recurrence by more than 3 times and concluding that this subgroup of patients should primarily be treated by radiotherapy and chemotherapy. Dalal found an overall survival rate of 92% at 5 years for patients with round cells < 5% compared to 74% of patients with round cells > 5% [25].

The role of chemotherapy in patients with soft tissue sarcomas has been extensively investigated [26] and several studies highlighted the potential sensitivity of liposarcoma to chemotherapy [23, 26–28]. Given the high risk of developing metastases, high grade MLs are suitable to chemotherapy and to new experimental protocols.

Tumoral site (upper limb, lower limb, and trunk) did not result in a significant risk factor, even though the few number of patients with trunk localization could have hampered statistical significance.

Tumor size is generally considered a prognostic factor for soft tissue sarcomas. Several studies have reported that larger tumors > 10 cm are associated with a poor prognosis [19, 20, 22, 29]. Size did not represent a significant prognostic factors in our series.

Local recurrence in our series was associated with an increased risk to develop metastases (*p* = 0.0437) and death due to cancer. Five patients who developed early local recurrence, simultaneously or subsequently developed metastases and all died. Early local recurrence is generally considered a poor prognostic indicator [19].

According to other authors, we observed a high rate of extrapulmonary metastases in MLs. Metastatic spread involved the lungs in 45% of cases and extrapulmonary sites in 55% of cases. Estourgie reported extrapulmonary metastases in 55% of patients with metastastic disease and recommended to follow up the patients with regular CT scan of the abdomen and pelvis [11]. Guadagnolo et al. reported 78% of metastasis localized in extrapulmonary sites, of which 48% in retroperitoneal space [23]. Several other authors found a high rate of extrapulmonary metastases in MLs, ranging from 41% to 77% [10, 12, 13, 18]. From these reports, common sites of metastases were the retroperitoneum, the abdominal and thoracic wall, and the abdominal cavity. Schwab et al. reported the skeleton as the most frequent site of metastasis, identifying 8 patients with skeletal lesions in a population of 184 MLs (4.3%). In this series, more than half of metastases (56%) were skeletal lesions, in particular localized to the spine, 70% in the absence of pulmonary localizations [8].

The reason of the tendency of MLs to metastatic spread in extrapulmonary sites is not clear. Ogose et al. speculated that the abundance of fat tissue in metastatic sites, such as the subcutaneous tissue, retroperitoneum, bone marrow, and the epidural space might favour the metastatic seeding [30].

An important issue is to assess whether extrapulmonary lesions are metastatic lesions or different sites of metachronous disease. Smith et al., analyzing the genomic rearrangement of TLS, CHOP, or EWS in six patients, confirmed the monoclonal origin of myxoid multifocal liposarcoma. They concluded that this unusual clinical phenomenon may represent a pattern of hematogenous metastatic spreading to other soft tissue sites, with cells unable to colonize the lungs [31].

Some authors highlighted an influence in the prognosis of some factors such as adipophilin, a well-known adipogenesis marker that appears early in the differentiation process [32], perhaps suggesting that MLs differentiate beyond the initial stage before the interruption of complete adipocyte maturation. Hoffmann et al. observed a significantly higher level of adipophilin in high grade than in low grade MLs, suggesting a role in the progression of the disease [33]. Other factors which are particularly expressed in MLs are the adipogenesis regulator PPAR*γ* [34] and CXCR4 (chemokine receptor), overexpressed in high grade tumors [35]. Overexpression of p53 in MLs [12] correlated with a poor chemotherapeutic response. The PDGFR-*β*expression in the MLs was found most frequently in metastatic forms (especially to bone) than in localized lesions [36].

An issue is what kind of imaging to use during follow-up of MLs for an early detection of extrapulmonary metastases. Some authors reported the failure of both PET scan and bone scan to detect metastases of myxoid liposarcoma [8]. Other options are total body CT and MRI who remain the most reliable screening tools. In particular, total body MRI may reveal the presence of extrapulmonary metastases at an early stage, when they are still not symptomatic, without radiation exposure.

## 9. Conclusion

Our study confirmed that inadequate surgical margins in MLs represent a significant risk factor to develop metastases (*p* = 0.001) with consequent negative influence on overall survival (*p* = 0.002). Surgical excision of MLs should be performed in specialized centers by experienced sarcoma surgeons. Inadequate primary treatment more frequently leads to local recurrence and metastasis (*p* = 0.0243). Local recurrences increase the risk to develop metastases (*p* = 0.0437) and metastatic event has a highly significant impact on overall survival (*p* < 0.001). Grading affects OS (*p* = 0,0479) and MFS (*p* = 0,0055). A multidisciplinary approach to MLs is recommended, considering combining surgery to radiation therapy and/or chemotherapy in selected cases. The awareness of the high incidence of extrapulmonary metastases, especially in fat-rich areas, should lead to clinical and imaging investigation such as total body MRI, aiming to an early diagnosis.

## Figures and Tables

**Table 1:** Main features.

Characteristics	N°	%
Patients	148	100

Presentation:		
Primary	103	70
Local recurrence	26	17
Radicalization	19	13

Grading:		
Low grade (<5% round cell)	100	68
High grade (>5% round cell)	48	32

Site:		
Lower limb	129	87
Upper limb	13	9
Trunk	6	4

Size:		
>10 cm	47	32
5–10 cm	67	45
<5 cm	34	23

**Table 2:** Surgical margins, radiotherapy, chemotherapy, local recurrence, and metastasis.

	Wide/radical	Marginal	Intralesional
Margin	105	41	2

	Preoperative		Postoperative
Radiotherapy	41		63

Chemotherapy	Neoadjuvant		Adjuvant
	25		29

Local recurrence	15 (10%)	8 (wide/radical surgery),	7 (marginal surgery)

Metastasis	20 (14%)	7 (wide/radical surgery),	15 (marginal surgery)

Site metastasis	55% extrapulmonary, 45% pulmonary (9 lung, 2 liver, 5 spine, 1 peritoneum, 1 kidney, 1 dorsal soft tissue, and 1 chest wall)		

**Table 3:** Statistical analysis indicates that margins (*p* = 0.002), grading (*p* = 0,0479), and the metastasis (*p* < 0,001) are risk factors on overall survival (OS).

Overall survival			
Variables	Survival at 5 years (%)	Survival at 10 years (%)	*p* value (LR test)
Site			
Upper limb	92	73	0,6215
Lower limb	89	86	
Trunk	100	100	

Size			
<5 cm	81	81	0,4268
5–10 cm	95	88	
>10 cm	89	89	

Grading, round cell (RC)			
Low (RC < 5%)	95	87	0,0479
High (RC > 5%)	80	80	

Margin			
Wide/radical	96	92	0,002
Marginal	76	66	
Intralesional	50	50	

Presentation			
Primitive	91	91	0,0755
Local Recurrence	78	72	
Radicalization	100	80	

LR			
No	92	86	0,2821
Yes	76	76	

RT			
No	90	90	0,7921
Yes	90	85	

CHT			
No	94	87	0,1766
Yes	83	83	

Metastases			
No	98	98	<0,0001
Yes	42	22	

**Table 4:** Statistical analysis shows no significant risk factors for the local recurrence-free survival (LRFS).

Local recurrence-free survival			
Variables	Survival at 5 years (%)	Survival at 10 years (%)	*p* value (LR test)
Site			
Upper limb	91	91	0,5852
Lower limb	89	85	
Trunk	67	67	

Size			
<5 cm	97	82	0,2883
5–10 cm	91	91	
>10 cm	77	77	

Grading, round cell (RC)			
Low (RC < 5%)	89	87	0,4824
High (RC > 5%)	87	83	

Margin			
Wide/radical	92	88	0,1085
Marginal	78	78	
Intralesional	100	100	

Presentation			
Primitive	87	85	0,2061
Local recurrence	86	78	
Radicalization	100	100	

RT			
No	94	81	0,9303
Yes	88	86	

CHT			
No	90	88	0,2035
Yes	85	81	

**Table 5:** Statistical analysis indicates margins (*p* = 0.0001), grading (0,0055), type of presentation (*p* = 0.0243), and local recurrence (0.0437) as risk factors on metastasis-free survival (MFS).

Metastases-free survival			
Variables	Survival at 5 years (%)	Survival at 10 years (%)	*p* value (LR test)
Site			
Upper limb	72	72	0,4542
Lower limb	86	82	
Trunk	100	100	

Size			
<5 cm	86	86	0,2716
5–10 cm	88	86	
>10 cm	80	70	

Grading, round cell (RC)			
Low (RC < 5%)	91	88	0,0055
High (RC > 5%)	72	68	

Margin			
Wide/radical	94	88	0,0001
Marginal	62	62	
Intralesional	100	100	

Presentation			
Primitive	90	86	0,0243
Local recurrence	65	65	
Radicalization	93	84	

LR			
No	88	84	0,0437
Yes	65	65	

RT			
No	82	82	0,9645
Yes	86	82	

CHT			
No	87	84	0,2363
Yes	81	76	

## References

[1] Fritchie K. J., Goldblum J. R., Tubbs R. R. (2012). The expanded histologic spectrum of myxoid liposarcoma with an emphasis on newly described patterns: Implications for diagnosis on small biopsy specimens. *American Journal of Clinical Pathology*.

[2] Fletcher C. D., Hogendoorn P., Mertens F. (2013). *WHO Classification of Tumours of Soft Tissue and Bone*.

[3] Lewis J. J., Leung D., Woodruff J. M., Brennan M. F. (1998). Retroperitoneal soft-tissue sarcoma: Analysis of 500 patients treated and followed at a single institution. *Annals of Surgery*.

[4] Linehan D. C., Lewis J. J., Leung D., Brennan M. F. (2000). Influence of Biologic Factors and Anatomic Site in Completely Resected Liposarcoma. *Journal of Clinical Oncology*.

[5] McCormick D., Mentzel T., Beham A., Fletcher C. D. M. (1994). Dedifferentiated liposarcoma: clinicopathologic analysis of 32 cases suggesting a better prognostic subgroup among pleomorphic sarcomas. *The American Journal of Surgical Pathology*.

[6] Panagopoulos L., Mandahl N., Mitelman F., Aman P. (1995). Two distinct FUS breakpoint clusters in myxoid liposarcoma and acute myeloid leukemia with the translocations t(12;16) and t(16;21). *Oncogene*.

[7] Pérez-Losada J., Sánchez-Martín M., Rodríguez-García M. A. (2000). Liposarcoma initiated by FUS/TLS-CHOP: The FUS/TLS domain plays a critical role in the pathogenesis of liposarcoma. *Oncogene*.

[8] Schwab J. H., Boland P., Guo T. (2007). Skeletal metastases in myxoid liposarcoma: An unusual pattern of distant spread. *Annals of Surgical Oncology*.

[9] Fletcher C. D. M., Unni K. K., Mertens F., Kleihues P. (2002). Atypical lipomatous tumour/Well differentiated liposarcoma, Dedifferentiated liposarcoma, Myxoidliposarcoma, Pleomorphic liposarcoma. *Pathology & Genetics, Tumours of Soft Tissue and Bone*.

[10] Cheng E. Y., Springfield D. S., Mankin H. J. (1995). Frequent incidence of extrapulmonary sites of initial metastasis in patients with liposarcoma. *Cancer*.

[11] Estourgie S. H., Nielsen G. P., Ott M. J. (2002). Metastatic patterns of extremity myxoid liposarcoma and their outcome. *Journal of Surgical Oncology*.

[12] Antonescu C. R., Tschernyavsky S. J., Decuseara R. (2001). Prognostic impact of P53 status, TLS-CHOP fusion transcript structure, and histological grade in myxoid liposarcoma: a molecular and clinicopathologic study of 82 cases. *Clinical Cancer Research*.

[13] Fiore M., Grosso F., Lo Vullo S. (2007). Myxoid/round cell and pleomorphic liposarcomas: prognostic factors and survival in a series of patients treated at a single institution. *Cancer*.

[14] Lemeur M., Mattei J.-C., Souteyrand P., Chagnaud C., Curvale G., Rochwerger A. (2015). Prognostic factors for the recurrence of myxoid liposarcoma: 20 cases with up to 8 years follow-up. *Orthopaedics & Traumatology: Surgery & Research*.

[15] Nishida Y., Tsukushi S., Nakashima H., Ishiguro N. (2010). Clinicopathologic prognostic factors of pure myxoid liposarcoma of the extremities and trunk wall. *Clinical Orthopaedics and Related Research*.

[16] Moreau L.-C., Turcotte R., Ferguson P. (2012). Myxoid∖round cell liposarcoma (MRCLS) revisited: An analysis of 418 primarily managed cases. *Annals of Surgical Oncology*.

[17] Haniball J., Sumathi V. P., Kindblom L.-G. (2011). Prognostic factors and metastatic patterns in primary myxoid/round-cell liposarcoma. *Sarcoma*.

[18] Ten Heuvel S. E., Hoekstra H. J., Van Ginkel R. J., Bastiaannet E., Suurmeijer A. J. H. (2007). Clinicopathologic prognostic factors in myxoid liposarcoma: A retrospective study of 49 patients with long-term follow-up. *Annals of Surgical Oncology*.

[19] Zagars G. K., Ballo M. T., Pisters P. W. T. (2003). Prognostic factors for patients with localized soft-tissue sarcoma treated with conservation surgery and radiation therapy: an analysis of 1225 patients. *Cancer*.

[20] Engström K., Bergh P., Gustafson P. (2008). Liposarcoma: outcome based on the Scandinavian Sarcoma Group register. *Cancer*.

[21] Chandrasekar C. R., Wafa H., Grimer R. J., Carter S. R., Tillman R. M., Abudu A. (2008). The effect of an unplanned excision of a soft-tissue sarcoma on prognosis. *The Journal of Bone and Joint Surgery—British Volume*.

[22] Kilpatrick S. E., Doyon J., Choong P. F. M., Sim F. H., Nascimento A. G. (1996). The clinicopathologic spectrum of myxoid and round cell liposarcoma: A study of 95 cases. *Cancer*.

[23] Guadagnolo B. A., Zagars G. K., Ballo M. T. (2008). Excellent Local Control Rates and Distinctive Patterns of Failure in Myxoid Liposarcoma Treated With Conservation Surgery and Radiotherapy. *International Journal of Radiation Oncology Biology Physics*.

[24] de Vreeze R. S. A., de Jong D., Haas R. L., Stewart F., van Coevorden F. (2008). Effectiveness of Radiotherapy in Myxoid Sarcomas Is Associated With a Dense Vascular Pattern. *International Journal of Radiation Oncology • Biology • Physics*.

[25] Dalal K. M., Kattan M. W., Antonescu C. R., Brennan M. F., Singer S. (2006). Subtype specific prognostic nomogram for patients with primary liposarcoma of the retroperitoneum, extremity, or trunk. *Annals of Surgery*.

[26] Patel S. R., Andrew Burgess M., Plager C., Papadopoulos N. E., Linke K. A., Benjamin R. S. (1994). Myxoid liposarcoma. Experience with chemotherapy. *Cancer*.

[27] Jones R. L., Fisher C., Al-Muderis O., Judson I. R. (2005). Differential sensitivity of liposarcoma subtypes to chemotherapy. *European Journal of Cancer*.

[28] Grosso F., Jones R. L., Demetri G. D. (2007). Efficacy of trabectedin (ecteinascidin-743) in advanced pretreated myxoid liposarcomas: a retrospective study. *The Lancet Oncology*.

[29] Pitson G., Robinson P., Wilke D. (2004). Radiation response: an additional unique signature of myxoid liposarcoma. *International Journal of Radiation Oncology • Biology • Physics*.

[30] Ogose A., Hotta T., Inoue Y., Sakata S., Takano R., Yamamura S. (2001). Myxoid liposarcoma metastatic to the thoracic epidural space without bone involvement: report of two cases. *Japanese Journal of Clinical Oncology*.

[31] Smith T. A., Easley K. A., Goldblum J. R. (1996). Myxoid/round cell liposarcoma of the extremities: A clinicopathologic study of 29 cases with particular attention to extent of round cell liposarcoma. *The American Journal of Surgical Pathology*.

[32] Heid H. W., Moll R., Schewetlick I., etal. (2012). Adipophilin is a specific marker of lipid accumation in diverse cell types and diseases. *Cell Tissue Res*.

[33] Hoffman A., Ghadimi M. P., Demicco E. G. (2013). Localized and metastatic myxoid/round cell liposarcoma. *Cancer*.

[34] Cheng H., Dodge J., Mehl E.

[35] Kim R. H., Li B. D., Chu Q. D. (2011). The Role of Chemokine Receptor CXCR4 in the Biologic Behavior of Human Soft Tissue Sarcoma. *Sarcoma*.

[36] Olofsson A., Willén H., Göransson M. (2004). Abnormal expression of cell cycle regulators in FUS-CHOP carrying liposarcomas. *International Journal of Oncology*.

